# Systematic Review Estimating the Burden of Dementia in the Latin America and Caribbean Region: A Bayesian Approach

**DOI:** 10.3389/fneur.2021.628520

**Published:** 2021-07-28

**Authors:** Yawen Xiang, Kimberly Vilmenay, Adrienne N. Poon, Shant Ayanian, Christopher F. Aitken, Kit Yee Chan

**Affiliations:** ^1^Centre for Global Health, Usher Institute, University of Edinburgh, Edinburgh, United Kingdom; ^2^Edinburgh Medical School, College of Medicine and Veterinary Medicine, University of Edinburgh, Edinburgh, United Kingdom; ^3^College of Medicine, Howard University, Washington, DC, United States; ^4^Department of Medicine, School of Medicine and Health Sciences, George Washington University, Washington, DC, United States; ^5^Department of Economics, Edinburgh Business School, Heriot-Watt University, Edinburgh, United Kingdom; ^6^Nossal Institute for Global Health, Melbourne School of Population and Global Health, University of Melbourne, Melbourne, VIC, Australia

**Keywords:** dementia, Latin America and the Caribbean (LAC), burden of dementia, low- and middle-income countries (LMICs), Bayesian approach

## Abstract

**Background:** The global burden of dementia has increasingly shifted to low- and middle-income regions that lack essential data for monitoring epidemiological progression, and policy and planning support. Drawing upon data that have emerged since the last known estimates published in 2015, this study aims to update dementia estimates in the Latin America and Caribbean (LAC) region for the years 2020, 2030, and 2050 through the application of a recently validated Bayesian approach for disease estimates useful when data sources are scarce.

**Methods:** A comprehensive parallel systematic review of PubMed, EMBASE, PsycINFO, Global Health, and LILACS was conducted to identify prospective population-based epidemiological studies on dementia published in English from 2013 to 2018 in LAC. English and non-English data cited by a recent review on dementia estimates in LAC were also examined for additional data. A Bayesian normal-normal hierarchical model (NNHM) was developed to estimate age-specific and age-adjusted dementia prevalence in people aged 60+. Using age-specific population projections from the UN, the total number of people affected by dementia for the years 2020, 2030, and 2050 were estimated.

**Results:** 1,414 studies were identified, of which only 7 met the inclusion criteria. The studies had 7,684 participants and 1,191 dementia cases. The age-standardized prevalence of all forms of dementia in LAC was 8% (95% CI: 5–11.5%) in people aged 60+. The estimated prevalence varied with age, increasing from 2.5% (95% CI: 0.08–4.0%) in the 60-69 age group, to 9.4% (95% CI: 5.4–13.2%) in the 70–79 age group and 28.9% (95% CI: 20.3–37.2%) in the ≥80 age group. The number of people age 60 and older living with dementia in LAC in 2020 was estimated at 6.86 (95% CI: 4.3–9.8) million, 9.94 (95% CI: 6.16–14.15) million in 2030, and 19.33 (95% CI: 12.3–13.6) million in 2050.

**Conclusion:** We project an upward disease trajectory for dementia in LAC countries. The projection is likely an underestimation of the true dementia burden given the underrepresentation of rural and socio-economically deprived populations. More research is urgently needed to improve the accuracy of disease estimates, guide clinicians to improve evaluations for earlier recognition of dementia, and support the development of effective policies for improving dementia prevention, diagnosis and clinical management in LAC's diverse and aging communities.

## Introduction

Dementia is an age-related neurocognitive disorder that has become a leading cause of morbidity and mortality in later life. In 2015, there were an estimated 47 million dementia cases globally, costing an estimated US$818 billion (1). With the number of dementia cases worldwide projected to double every 20 years, this cost is estimated to rise to US$2 trillion by 2030. The increasing majority of this burden has been shifting to low- and middle-income countries (LMICs) that have been undergoing drastic demographic and health transitions. In Latin America and the Caribbean (LAC), the average life expectancy has increased by almost 20 years since 1960 (2). The population aged 60 years and over grew by 6.6 times during this period (from 12.7 to 84.9 million) and is projected to increase to 190 million by 2050 (3). As the population experiences longer health expectancy than prior generations, the risk of dementia and prevalence may both increase.

Previous studies that estimated the prevalence of dementia in LAC have been conducted as part of larger multi-regional dementia prevalence estimates (1, 4–6). An issue with using a global model for estimating regional estimates is that countries with better health informatics infrastructures tend to have more complete data and may disproportionately influence the model, and shield the LAC specific data from being relevant. Furthermore, using LAC specific data would account for population variability in terms of genomics, quality of life and education, which are known to affect dementia, thus making the conclusions stronger in terms of applicability for the LAC area. The lack of a standalone model that can estimate the burden of dementia based on LAC data is perhaps due to the historical dearth of data in LMICs. The most recent replicable systematic review of PubMed identified only 14 quality studies published between 1990 and 2013 that contained original data on the prevalence of dementia in LAC (1). Moreover, that meta-analysis estimated a high prevalence of 8.34% for people aged 60 and over – second only to North Africa/Middle East (8.7%). This high prevalence poses a considerable challenge to health and economic systems which lack both the ability to adequately diagnose dementia but also the availability of public and private grants in order to do so (7). The evidence for dementia in LAC is likely to have expanded since this prior estimate. An updated review and estimates are thus timely and pertinent.

To make the best use of the scarce data, in recent years, modelers have turned away from the traditional frequentist analytic approach in favor of Bayesian methods which are considered more suitable for meta-analyzing small datasets [k ≥ 2 (6)]. By allowing historical data (prior estimates) to be incorporated into current estimates (8, 9), Bayesian methods also improve the consistency between current and prior estimates (10). Establishing an optimal approach for applying Bayesian methods to prevalence estimates is an area of ongoing study. To this end, Poon et al. (11) recently updated estimates of the burden of dementia for the South-East Asia region using two Bayesian approaches and confirmed the estimates using a traditional frequentist approach. The study showed that results using all three approaches were comparable, though Bayesian stands as a more promising methodology for improving estimates for severely limited datasets. One of these approaches, the Bayesian Bayesmeta algorithm, uses a newer, simpler and open source software that could give opportunities for researchers in low resource settings to participate in disease estimates without paying software subscriptions (12). Given the novelty of this approach, results would benefit from further validation.

The overall aim of this study is to provide an updated estimate of the prevalence of dementia for LAC with improved accuracy. By way of a comprehensive systematic review of a larger number of academic and non-academic databases, we aim to identify data that has emerged from the LAC region since 2013. The total number of dementia cases in LAC will be estimated for the years 2020, 2030, and 2050. The study will also explore the newer Bayesian Random-Effects Meta-Analysis (Bayesmeta) in R (13) against the more established Bayesian JAGS algorithm (14). It is hoped that the updated estimates will help draw attention to the growing burden of dementia in LAC as part of a global trend. This study can generate evidence-based burden estimates that are key for informing policy and healthcare-planning and a knowledge base to support clinicians for earlier identification and management of dementia.

## Methods

### Search Strategy

Systematic parallel searches were conducted by YX and KV using PubMed, EMBASE, Global Health, PsycINFO and LILACS, and the gray literature. In order to capture the broadest number of studies during the search process, we used the United Nations M49 standard definition of LAC which provides a broad definition of the geographical region of LAC (15). This includes all countries south of the United States, West of the Atlantic Ocean, east of the Pacific Ocean, and north of Antarctica; resulting in a total of 52 countries. The overall search terms were “(Dementia or Alzheimer^*^) AND (prevalence OR incidence OR morbidity OR mortality OR “burden of disease” OR “disease burden” OR Epidemiology) AND (“Latin America” OR Caribbean OR “Central America” OR [names of each included countries separated by an “OR”]) adapted to the syntax requirements of the specific database (see Supplementary Material 1 for details). Google Scholar and hand searches were used to identify any relevant gray literature. Additionally, non-English data cited in a recent review by Nitrini et al. were hand-searched for additional data (7).

### Inclusion and Exclusion Criteria

We included only studies that: (i) were prospective and population-based; (ii) contained original data on incidence, prevalence and/or mortality of dementia; (iii) used internationally recognized diagnosis of dementia [i.e., Diagnostic and Statistical Manual of Mental Disorders (DSM) (16) or International Classification of Diseases criteria for dementia (ICD) (17), National Institute of Neurological and Communicative Disorders and Stroke and the Alzheimer's Disease and Related Disorders Association criteria for Alzheimer's disease (NINCDS-ADRDA) (18), National Institute of Neurological Disorders and Stroke and Association Internationale pour la Recherché et l'Enseignement en Neurosciences criteria for vascular dementia (NINDS-AIREN) (19) and the 10/66 Dementia Diagnostic Algorithm] (20); and (iv) published between 2013 and December 2018.

Within the systematic review process from the databases, we excluded: (i) duplicates within and between the databases; (ii) studies with no original numerical estimates (e.g., reviews, viewpoints); (iii) studies of LAC populations outside of LAC; (iv) non-human studies; (v) studies with no clear denominator or inappropriate standardized rates; (vi) studies of non-community-based populations (e.g., nursing homes) and (vii) non-English language studies.

### Quality Assessment

Quality assessment was conducted by YX and KV using a modified version of the Joanna Briggs Institute (JBI) Critical Appraisal checklist for prevalence studies (Supplementary Material 2).

### Data Extraction and Analysis

For each eligible study, the following data were extracted: (i) study country of origin; (ii) sampling method; (ii) screening tool(s); (iii) diagnostic tool(s); (iv) sample size (denominator); and (v) the number of dementia cases (numerator) and/or unweighted dementia prevalence. Where available, we also extracted incidence data, mortality data, prevalence data by urban vs. rural population, age group, gender and types of dementia.

Using the Bayesmeta package of R (version 3.5.2) (12, 13), a Bayesian normal-normal hierarchical model (NNHM) was used to estimate age-specific and age-adjusted dementia prevalence in people aged 60 and above. The prior prevalence estimates published by Prince and Wimo (1) for the age groups 60–69, 70–79, and 80 and over were used in this model, alongside the newly extracted data from the current study.

The foremost step was to sort the number of screened participants and the number of people with dementia (PWD) identified from each study into 10-year group bins. Any participant over the age of 80 was allocated into an “80 and over” bin. Our variance was then set to 0.09^2^, 0.15^2^, and 0.3^2^ for age groups 60–69, 70–79, and 80 and over, respectively. To account for the variability in prevalence estimates with increasing age, the variance was widened in each subsequent group due to the reduction in sample sizes in the older age groups. Prevalence was then pooled for groups 60–69, 70–79, and 80 and over, and 95% credible intervals were calculated.

To test the sensitivity of the Bayesmeta package, we used the Just Another Gibbs Sampler (JAGS) (14), an open source algorithm used often in Bayesian analysis, to generate 3 Markov chain Monte Carlo (MCMC) chains. These MCMC chains produced disease burden estimates for each of the allotted age group bins highlighted above using a similar NNHM model. Using age-specific population projections from the United Nations Development Program (UNDP) (3), the total number of people affected by dementia for the years 2020, 2030, 2050 were estimated.

## Results

**Figure 1:**
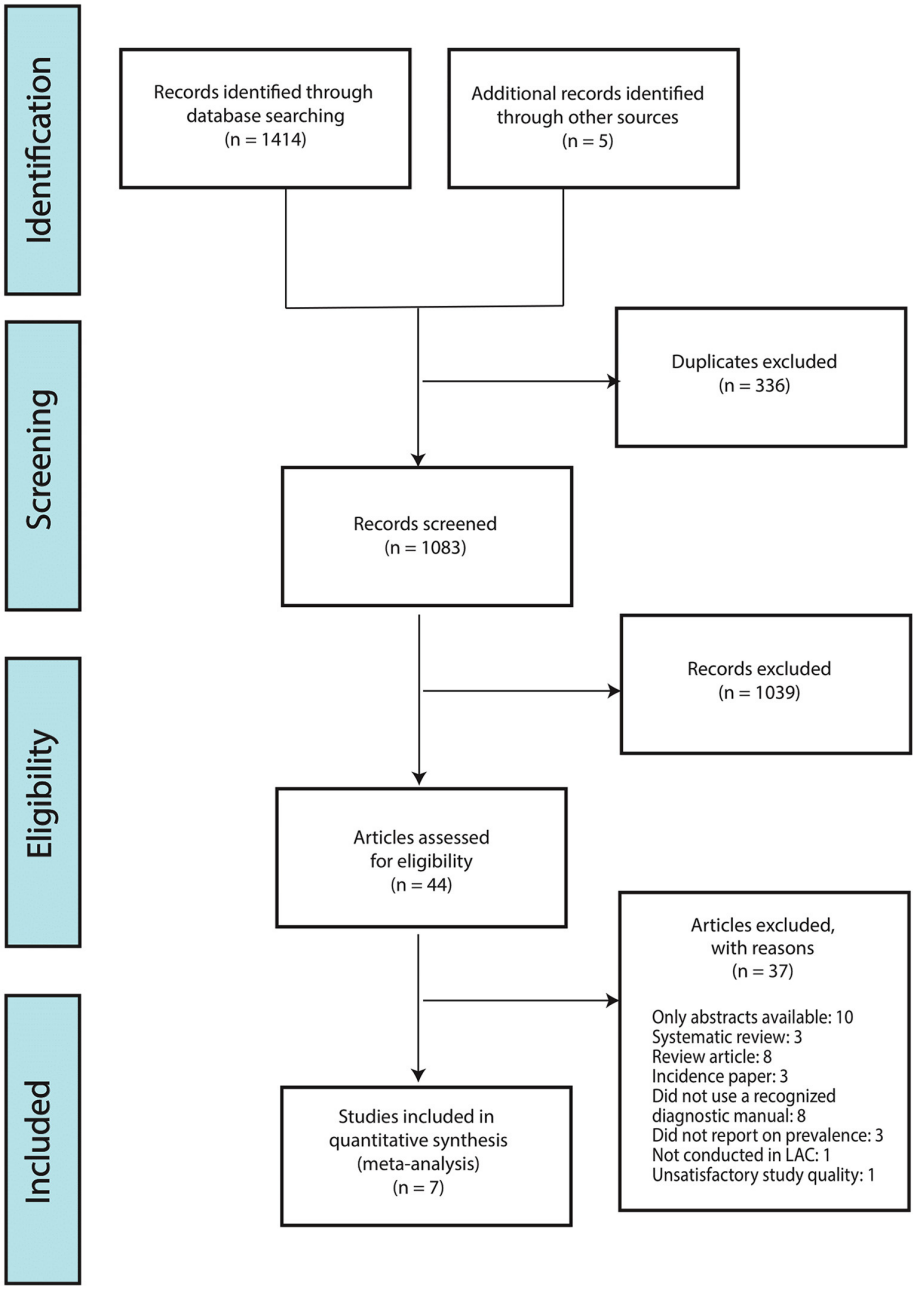
PRISMA of study selections.

The searches yielded a total of 1,414 articles; 1,082 after removing duplicates. Of the remaining articles 1,041 articles were further excluded based on the title and abstract relevance and 41 full-text articles were then analyzed based on our set inclusion/exclusion criteria, study design and use of case definitions. Of these, only 7 studies met all inclusion criteria and were retained for meta-analysis (see Supplementary Material 2 for quality assessment using JBI). Our reading of Nitrini et al.'s recent review on the current trends and challenges of dementia in LAC (7) yield two systematic reviews that incorporated dementia data from LAC in English and Spanish; i.e., Nitrini et al. (21) and Sanchez et al. (22). Neither of these reviews yielded further studies that met our inclusion criteria (see Supplementary Material 3 for detail). The number of participants in the retained studies was 7,684 with all studies having recruited more female participants than male participants: the proportion of female participants ranged from 55.8 to 74.4%. The range of participants in the included studies varied from 301 participants (23) to 1,898 (24). Figure 1 illustrates the selection process.

As Table 1 illustrates, the majority of the studies (four) were conducted in South America (25, 26, 29), two were based in the Caribbean (23, 24), and one in Central America (27). With the exception of the Central American study, all studies had adopted a 2-stage design that involved initial screening by field workers and case confirmation by medical specialists (see Table 1). All studies reported the prevalence of dementia and were cross-sectional in design. None of the studies reported incidence or mortality of dementia. Supplementary Materials 4 provides results of our assessment that shows the retained studies are of high quality.

**Table 1:** Characteristics of the retained studies.

**References**	**Region/Country**	**Sample size and Response rate (%)**	**Participants traits**	**Sample selection**	**Participant recruitment**	**Study design**	**Screening tools**	**Outcome ascertainment**
Davis et al. (24)	Trinidad, Caribbean	2,378 approached 1,898 analyzed (79.8%)	44% Male 56% Female >70 years old	Comprehensive cross-sectional survey from nationally representative sample	Random and proportional sampling	Single-phase cross-sectional survey	10/66 Community Screening Instrument for Dementia	10/66 short dementia diagnostic algorithm
Eldemire-Shearer et al. (23)	Jamaica, Carribean	340 approached 301 analyzed (88.5%)	42.5% Male 57.5% Female >60 years old	National representative cross-sectional survey	Random sampling of 340 from a nationally representative cohort	2-stage design cross-sectional study^*^	MMSE	DSM-IV MRI classification
Pedraza et al. (25)	Bogotá, Columbia, South America	1,263 approached 1235 analyzed (97.8%)	25.6% Male 74.4% Female >50 years old	Community dwelling elderly population	Consecutive sampling	2-stage design cross-sectional study^*^	MMSE SMCQ MoCA	DSM-IV
Cesar et al. (26)	Tremembé, Brazil, South America	738 approached 630 analyzed (85.4%)	37% Male 63% Female >60 years old	Representative of the region in terms of socioeconomic and cultural levels	Random sampling	Single-phase cross-sectional survey	MMSE BCSB	NIA-AA
Bartoloni et al. (27)	Suburban area of Buenos Aires, Argentina, South America	2,437 approached 1795 analyzed (73.7%)	44.2% Male 55.8% Female >60 years old	Seven slums in Matanza Riachuelo	Consecutive sampling by door to door survey	2-stage design cross-sectional study^*^	MMSE	DSM-IV
Velázquez-Brizuela et al. (28)	Metropolitan area of Guadalajara, Jalisco, Mexico, Central America	1,142 analyzed (NA)	36.2% Male 63.8% Female >60 years old	Representative of the region	Multistage and proportional random sampling	2-stage design cross-sectional study^*^	MMSE	DSM-IV
Correa Ribeiro et al. (29)	Rio de Janeiro, Brazil, South America	769 eligible 736 screened 683 analyzed (95.7%)	29.1% Male 70.9% Female >65 years old	Clients of private health care plan, older than 65 years of age	Random sampling from client pool	2-stage design cross-sectional study^*^	MMSE	DSM-IV, ICD-10, NINCDS-ADRDA, NINDS-AIREN

* *Two-stage cross-sectional study involving: 1. Screening (MMSE) by trained fieldworkers; and 2. Case confirmation by psychiatrists*.

*Mini-Mental State Examination (MMSE); Montreal Cognitive Assessment (MoCA); Subjective memory complaints questionnaire (SMCQ); Brief Cognitive Screening Battery (BCSB); the International Classification of Diseases (ICD); the Diagnostic and Statistical Manual of Mental Disorders (DSM); National Institute on Aging and Alzheimer's Association also published diagnostic guidelines (NIA-AA); National Institute of Neurological Disorders and Stroke Association criteria for vascular dementia (NINDS-AIREN); National Institute of Neurological and Communicative Disorders and Stroke and the Alzheimer's Disease and Related Disorders Association criteria for Alzheimer's disease (NINCDS-ADRDA)*.

### Prevalence Estimates

Based on Bayesian NNHM estimates, the prevalence of dementia for people aged 60 and above in LAC was 8% (95% Credible Interval: 5–11.5%). As expected, the prevalence of dementia increased with age, from 2% (1–4%) for the 60–69 age group to almost 30% (29%, 20–37%) for the 80 and over age group (Table 2). Apart from the 70–79 age group, our prevalence estimates are closely comparable to those of Prince and Wimo (1). The sensitivity test using the classical MCMC found similar results, supporting the validity of our estimates (Supplementary Material 5).

**Table 2:** Pooled age-specific and age-standardized prevalence and number of PWD in LAC for the years 2015, 2020, 2025, 2030, and 2050.

	**Pooled prevalence (CI 95%)**		**Number of PWD in LAC from our estimates (thousands)**		
**Age**	**Prince and Wimo (1)**	**Our estimates**	**2020**	**2030**	**2050**
60–69	0.02	0.02 (0.01–0.04)	942 (471–1,884)	1,239 (620–2,478)	1,729 (864–3,457)
70–79	0.07	0.09 (0.05–0.13)	2,266 (1,259–3,273)	3,399 (1,889–4,910)	5,591 (3,106–8,076)
≥80	0.21	0.29 (0.20–0.37)	3,647 (2,515–4,653)	5,299 (3,654–6,761)	12,014 (8,286–15,329)
≥60	0.08	0.08 (0.05–0.115)	6,855 (4,245–9,811)	9,938 (6,163–14,149)	19,334 (12,256–26,862)

*ADI (2015) provided prevalence estimates in 5-year age groupings. In this study, the mean of two age groups were taken to represent the prevalence estimates in 10-year age groupings (e.g., the prevalence was 0.015 for people aged 60–64 and 0.026 for people aged 65–69, therefore the prevalence of people aged between 60–69 was determined to be the mean of the two estimates, 0.0205.). The ADI 2015 estimates were used as a comparison for our Bayesian estimation*.

*Pooled prevalence from ADI (2015) are provided here for comparison*.

Of the seven studies, two reported breakdowns of prevalence by sex (26, 29). No studies reported prevalence by rural vs. urban areas, and two provided prevalence breakdowns by dementia subtypes (23, 29). Unfortunately, their combined sample sizes were too small to allow analysis by any of these variables.

### Burden Estimates

By applying the UNDP population projection (3), the number of dementia cases for people aged 60 and above in LAC is estimated at 6.86 (95% Credible Interval: 4.3–9.8) million, 9.94 (CI: 6.16–14.15) million in 2030, and 19.33 (CI: 12.3–13.6) million in 2050. Table 2 provides the breakdown of the case number by 10-year age groups for the years 2020, 2030, and 2050. As expected, people over the age of 80 had the highest burden of dementia, from 3.6 (CI: 2.5–4.7) million in 2020 to 5.3 (CI: 3.7–6.8) million in 2030 to 12 (CI: 8.3–15.3) million in 2050. This age group is also predicted to take up an increasing proportion of the overall dementia cases from 53% in 2020 and 2030, to 62% in 2050.

## Discussion

This study updated the estimates of the burden of dementia in LAC using the latest epidemiological data from the region. To our knowledge, this is the first comprehensive systematic review that has exclusively focused on modeling the prevalence of dementia in LAC. Our results confirmed a high dementia prevalence of 8% in LAC, and a substantial (2.8-fold) increase in the number of dementia cases over the next three decades. The results also indicate that an increasing majority of the cases will fall upon the oldest (≥80) age group. This will place tremendous strains on the already fragile local health and social systems, as well as families and individuals.

While the cost of dementia might seem low in monetary terms in LMICs relative to HICs, the lack of publicly funded formal assistance means the majority of the cost is borne by families (1, 30). Women often bear the role of the informal caregivers both within the family as well as in hired help. In Brazil, for instance, the cost of informal care (including costs associated with carers' loss in economic productivity) represents approximately three times the minimum wage, making it out of reach for the majority of people with dementia (7). The increase in the proportion of dementia cases in the oldest age group is also particularly problematic in light of decreasing family sizes, especially in urban areas (31). Elderly living with dementia often suffers from a number of associated comorbid conditions, requiring an increase in the number of providers, specialized care and healthcare spending to meet the unique clinical challenges of this group. Further burdens may include additional medications, assistance with activities of daily living including feeding, reductions in mobility, and pressure sores. Moreover, an increase in dementia cases in this age group could see an increase in the burden of care falling upon other elderly family members who themselves require assistance. Individuals living with severe dementia may rely more heavily on healthcare in long-term care facilities and eventually hospice (7). The infrastructure and support for such facilities may need policy makers to consider their expansion to meet demands for an aging population with dementia. Additional support to train providers and community members is needed for early identification of dementia. The role of interventions is also largely unknown, but available treatment options may be useful in earlier stages.

Alarmingly, the high prevalence reported in our study is a likely underestimation of the true prevalence of dementia in LAC. Risk factors of dementia, including illiteracy, low educational attainment, hypertension, obesity and diabetes, have been shown to disproportionately affect socio-economically deprived populations (32). LAC is one such region markedly affected by widespread socio-economic inequalities. Eleven LAC countries are amongst the top 30 nations with the worst Gini scores (33), while an estimated 23.3% of the population still live under poverty [<$5.50 a day (34)]. Illiteracy and low education attainment, in particular, are key drivers of dementia in LAC (7). One study in LAC found that the prevalence of dementia may be doubled in the illiterate population relative to the literate population (20). Illiteracy is particularly prevalent amongst LAC's elderly population (21.1%) (35), especially in indigenous populations and populations in rural areas (7). Yet, these populations are not represented in the studies identified for our current model, and are likely to have skewed our results toward an underestimation of the true burden of dementia in LAC.

The lack of data representativeness is largely a manifestation of the lack of research investment in the region resulting in data scarcity. Despite the comprehensiveness of our searches, only seven new cross-sectional studies from six of LAC's 52 countries/territories were identified. These studies together form a small sample of <8,000 people for a region with more than 70 million people over the age of 60 (3). Data scarcity is not an issue unique to the LAC region, but applies to most LMIC regions. One of the most comprehensive global dementia prevalence reviews did not identify any primary study from Central Europe, Australasia, South Asia and Southeast Asia published after 2010 (1). Another well-conducted global review included no study published after 2010 from Eastern Europe, Central Europe, Central Asia and Oceania. In the same review, only four studies published after 2010 were included for the estimate for the Middle East and Africa (6). The only known exception was our systematic review and meta-analysis on the epidemiology of dementia in China (1990–2010) using Chinese databases (i.e., CNKI and WanFang) (36). Our review returned 12,642 publications, of which 89 studies met the inclusion criteria. In total, 340,247 participants were assessed, and 9,900 were diagnosed with dementia. However, even with this relatively large number of studies, the research was still skewed toward urban and more developed parts of China, and did not sufficiently represent the diverse population of the country.

The lack of research investment also reflects a missed opportunity to capitalize on the unique populations (genetic clusters, low literacy, multilingual, multi-ethnic) offered by LAC, which could significantly enhance our understanding of the roles various population characteristics could play in the progression and risk factors of disease development (37). In addition, the lack of longitudinal studies that monitor incidence, mortality and the environmental and biological risk factors of dementia in the region complicates efforts to adequately understand the rising dementia cases in the region for developing contextually appropriate ways to effectively reduce risks.

A number of strengths and limitations of this study merits further mention. We have aimed to validate the Bayesmeta approach for data projections in the setting of data scarcity in this as well as our prior work (11). However, despite this paper being the second attempt at validating the Bayesmeta package with a separate JAGS algorithm, we have not contrasted the results of the two algorithms using inferential statistics. Furthermore, performing a systematic validation of the Bayesmeta package for use in meta-analysis is also needed, but is outside the scope of this paper. Due to limited resources, we were unable to conduct a full systematic review of non-English publications that contain dementia estimates in the LAC region – a limitation also observed in previous systematic reviews (1, 4) that have produced the currently accepted dementia estimates of LAC for the Alzheimer' Disease International (ADI) and the WHO. This limitation could have potentially reduced the number of studies identified for the region. To reduce the number of non-English studies we might have missed, we evaluated the studies cited by a recent review of dementia in LAC by Nitrini et al. (7) which included the two systematic reviews of English and non-English studies on the subject. Despite the 4-year overlap these reviews and the current study, no new data that met our inclusion criteria were identified. This gives us the confidence that any non-English studies that we might have missed are restricted to the 2-year period between 2017 and 2018. To give an indication on how many papers we might have missed for this 2-year period, we calculated the average number of non-English papers published per year that met our inclusion criteria to be 0.12 (see Supplementary Material 3). Thus, any data we might have missed is likely to be marginal and unlikely to have affected our current estimates. To further minimize the chance of missing valuable data, future reviews should strive to incorporate non-English studies when possible. Another limitation of our study is that our analyses were confined by the limited data reported in the studies used for our model. For example, the absence of case breakdowns by sex and dementia subtypes in the original papers has prevented the generation of sex-specific and subtype-specific estimates. This is a well-known issue in the burden of disease area. To maximize the value of the scarcely funded research, future publications of epidemiological studies on dementia should adopt standardized guidelines specifically designed to ensure more precise, consistent and transparent reporting. The adoption of two guidelines would also greatly assist global health researchers to better appraise the quality of studies, extract more relevant information and improve regional burden estimates (38–40). The first is the *Strengthening the Reporting of Observational Studies in Epidemiology (STROBE)* guidelines developed in 2007 for the accurate and complete reporting of observational epidemiological studies (38, 39). The second is the *Standards of Reporting of Neurological Disorders (STROND) Checklist*, in which Bennett et al. (40) which provides future guidance on the steps toward full and consistent reporting of neuro-epidemiological studies for the purpose of the burden of disease-type studies such as the current study.

## Conclusion

The results of this systematic review suggest that the LAC region is on an upward disease trajectory in terms of burden of dementia. There is a need for more dementia and dementia subtype epidemiological research in this region, especially from the less resourceful countries, in order to accurately estimate dementia prevalence and the health needs of a varied and diverse population. There is a need for greater globalization of knowledge with a greater emphasis placed on the amount and quality of evidence produced. With the trend of demographic aging in the coming decades, the prevalence and burden of dementia will continue to increase. This will have serious implications for the economy, healthcare systems and the communities in LAC. In particular, there may be additional burdens placed on caregivers and strain on healthcare facilities to meet demands of an aging population. Despite the comprehensive scope of our review, rural and socio-economically deprived populations, including indigenous, illiterate and low-literacy populations were underrepresented in these data. This is likely to have skewed our results toward underestimating the true burden in this region. Such information is paramount for guiding clinical practice, which will allow not only for improved evaluations in early dementia diagnosis but also guide the development of effective policies for improving dementia prevention, diagnosis and clinical management in LAC's aging communities.

## Data Availability Statement

The original contributions generated for the study are included in the article/Supplementary Material, further inquiries can be directed to the corresponding author/s.

## Author Contributions

KC and YX conceptualized the paper. YX and KV carried out the systematic review. YX, CA, and SA performed the analytic calculations. YX, KV, AP, and KC wrote the manuscript with input from all authors. All authors have discussed the results and reviewed the manuscript.

## Conflict of Interest

The authors declare that the research was conducted in the absence of any commercial or financial relationships that could be construed as a potential conflict of interest.

## Publisher's Note

All claims expressed in this article are solely those of the authors and do not necessarily represent those of their affiliated organizations, or those of the publisher, the editors and the reviewers. Any product that may be evaluated in this article, or claim that may be made by its manufacturer, is not guaranteed or endorsed by the publisher.
