# Supplementary material for: Systematic Review Estimating the Burden of Dementia in the Latin America and Caribbean Region: A Bayesian Approach
Source: Front Neurol. 2021 Jul 28;12:628520. doi: 10.3389/fneur.2021.628520 (PMC8356078; doi:10.3389/fneur.2021.628520)
Supplement: Supplementary file 1 [file Data_Sheet_1.docx]

Supplementary Materials

### Supplementary Materials 1 Search Terms:

| **Database** | **Search Syntax** |
| --- | --- |
| **Pubmed** | (Prevalen* OR inciden* OR epidemiolog* OR morbidity OR mortality OR "burden of disease" OR "disease burden") AND (dementia OR Alzheimer*) AND ("Latin America" OR "Caribbean" OR LAC OR Anguilla OR Antigua and Barbuda OR Aruba OR Bahamas OR Barbados OR Bonaire OR Sint Eustatius and Saba OR British Virgin Islands OR Cayman Islands OR Cuba OR Curacao OR Dominica OR Dominican Republic OR Grenada OR Guadeloupe OR Haiti OR Jamaica OR Martinique OR Montserrat OR Puerto Rico OR Saint Barthelemy OR Saint Kitts and Nevis OR Saint Lucia OR Saint Martin OR Saint Vincent and the Grenadines OR Sint Maarten OR Trinidad and Tobago OR Turks and Caicos Islands OR United states virgin islands OR Belize OR Costa Rica OR El Salvador OR Guatemala OR Honduras OR Mexico OR Nicaragua OR Panama OR Argentina OR Bolivia OR Bouvet Island OR Brazil OR Chile OR Colombia OR Ecuador OR Falkland Islands OR French Guiana OR Guyana OR Paraguay OR Peru OR South Georgia and South Sandwich Islands OR Suriname OR Uruguay OR Venezuela) AND ( ( "2013/01/01"[PDat] : "2018/12/31"[PDat] ) ) |
| **EMBASE** | prevalence/ or Prevalen*.mp.  incidence/ or inciden*.mp.  morbidity.mp. or morbidity/  mortality.mp. or mortality/  burden of disease.mp.  disease burden.mp. or disease burden/  epidemiology/ or epidemiolog*.mp.  dementia.mp. or dementia/  alzheimer.mp. or Alzheimer disease/  latin america.mp. or "South and Central America"/  Caribbean/ or caribbean.mp.  Anguilla.mp.  (Antigua and barbuda).mp. [mp=title, abstract, heading word, drug trade name, original title, device manufacturer, drug manufacturer, device trade name, keyword, floating subheading word, candidate term word]  Aruba/ or Aruba.mp.  bahamas.mp. or Bahamas/  barbados.mp. or Barbados/  bonaire.mp. or Bonaire/  British Virgin Islands.mp. or "Virgin Islands (British)"/  Cayman Islands.mp. or Cayman Islands/  Cuba.mp. or Cuba/  Curacao.mp. or Curacao/  Dominica.mp. or Dominica/  Dominican Republic.mp. or Dominican Republic/  Grenada.mp. or Grenada/  Guadeloupe.mp. or Guadeloupe/  Haiti.mp. or Haiti/  Jamaica.mp. or Jamaica/  Martinique.mp. or Martinique/  Montserrat/ or Montserrat.mp.  Puerto Rico.mp. or Puerto Rico/  Saint Barthelemy.mp. or Saint Barthelemy/  (Saint Kitts and Nevis).mp. [mp=title, abstract, heading word, drug trade name, original title, device manufacturer, drug manufacturer, device trade name, keyword, floating subheading word, candidate term word]  Saint Lucia.mp. or Saint Lucia/  Saint Martin.mp. or Saint Martin/  (Saint Vincent and the Grenadines).mp. [mp=title, abstract, heading word, drug trade name, original title, device manufacturer, drug manufacturer, device trade name, keyword, floating subheading word, candidate term word]  Sint Maarten.mp. or "Saint Martin (Dutch)"/  (Trinidad and Tobago).mp. [mp=title, abstract, heading word, drug trade name, original title, device manufacturer, drug manufacturer, device trade name, keyword, floating subheading word, candidate term word]  (Turks and Caicos).mp. [mp=title, abstract, heading word, drug trade name, original title, device manufacturer, drug manufacturer, device trade name, keyword, floating subheading word, candidate term word]  United States virgin islands.mp. or "Virgin Islands (U.S.)"/  Belize.mp. or Belize/  Costa Rica.mp. or Costa Rica/  El Salvador.mp. or El Salvador/  Guatemala.mp. or Guatemala/  Honduras.mp. or Honduras/  Mexico/ or Mexico.mp.  Nicaragua.mp. or Nicaragua/  Panama/ or Panama.mp.  Argentina.mp. or Argentina/  Bolivia.mp. or Bolivia/  Bouvet Island.mp. or Bouvet Island/  Brazil/ or Brazil.mp.  Chile.mp. or Chile/  Colombia.mp. or Colombia/  Ecuador.mp. or Ecuador/  Falkland Islands.mp. or "Falkland Islands (Malvinas)"/  French Guiana.mp. or French Guiana/  Guyana.mp. or Guyana/  Paraguay.mp. or Paraguay/  peru.mp. or Peru/  (South Georgia and the South Sandwich Islands).mp. [mp=title, abstract, heading word, drug trade name, original title, device manufacturer, drug manufacturer, device trade name, keyword, floating subheading word, candidate term word]  Suriname.mp. or Suriname/  Uruguay.mp. or Uruguay/  Venezuela.mp. or Venezuela/  1 or 2 or 3 or 4 or 5 or 6 or 7  8 or 9  10 or 11 or 12 or 13 or 14 or 15 or 16 or 17 or 18 or 19 or 20 or 21 or 22 or 23 or 24 or 25 or 26 or 27 or 28 or 29 or 30 or 31 or 32 or 33 or 34 or 35 or 36 or 37 or 38 or 39 or 40 or 41 or 42 or 43 or 44 or 45 or 46 or 47 or 48 or 49 or 50 or 51 or 52 or 53 or 54 or 55 or 56 or 57 or 58 or 59 or 60 or 61 or 62 or 63  64 and 65 and 66  limit 67 to (english language and yr="2013 -Current") |
| **PsycINFO** | Prevalen*.mp.  inciden*.mp.  morbidity.mp. or exp MORBIDITY/  mortality.mp.  exp Epidemiology/ or epidemiolog*.mp.  burden of disease.mp.  disease burden.mp.  exp DEMENTIA/ or dementia.mp.  exp ALZHEIMER'S DISEASE/ or alzheimer.mp.  latin america.mp.  caribbean.mp.  south america.mp.  central america.mp.  Anguilla.mp.  (Antigua and barbuda).mp. [mp=title, abstract, heading word, table of contents, key concepts, original title, tests & measures]  Aruba.mp.  bahamas.mp.  barbados.mp.  bonaire.mp.  (Sint Eustatius and Saba).mp. [mp=title, abstract, heading word, table of contents, key concepts, original title, tests & measures]  British Virgin Islands.mp.  Cayman Islands.mp.  Cuba.mp.  Curacao.mp.  Dominica.mp.  Dominican Republic.mp.  Grenada.mp.  Guadeloupe.mp.  Haiti.mp.  Jamaica.mp.  Martinique.mp.  Montserrat.mp.  Puerto Rico.mp.  Saint Barthelemy.mp.  (Saint Kitts and Nevis).mp. [mp=title, abstract, heading word, table of contents, key concepts, original title, tests & measures]  Saint Lucia.mp.  Saint Martin.mp.  (Saint Vincent and the Grenadines).mp. [mp=title, abstract, heading word, table of contents, key concepts, original title, tests & measures]  Sint Maarten.mp.  (Trinidad and Tobago).mp. [mp=title, abstract, heading word, table of contents, key concepts, original title, tests & measures]  (Turks and Caicos).mp. [mp=title, abstract, heading word, table of contents, key concepts, original title, tests & measures]  United States virgin islands.mp.  Belize.mp.  Costa Rica.mp.  El Salvador.mp.  Guatemala.mp.  Honduras.mp.  mexico.mp.  Nicaragua.mp.  Panama.mp.  Argentina.mp.  Bolivia.mp.  Bouvet Island.mp.  Brazil.mp.  Chile.mp.  Colombia.mp.  Ecuador.mp.  Falkland Islands.mp.  French Guiana.mp.  Guyana.mp.  Paraguay.mp.  Peru.mp.  (South Georgia and the South Sandwich Islands).mp. [mp=title, abstract, heading word, table of contents, key concepts, original title, tests & measures]  Suriname.mp.  Uruguay.mp.  Venezuela.mp.  1 or 2 or 3 or 4 or 5 or 6 or 7  8 or 9  10 or 11 or 12 or 13 or 14 or 15 or 16 or 17 or 18 or 19 or 20 or 21 or 22 or 23 or 24 or 25 or 26 or 27 or 28 or 29 or 30 or 31 or 32 or 33 or 34 or 35 or 36 or 37 or 38 or 39 or 40 or 41 or 42 or 43 or 44 or 45 or 46 or 47 or 48 or 49 or 50 or 51 or 52 or 53 or 54 or 55 or 56 or 57 or 58 or 59 or 60 or 61 or 62 or 63 or 64 or 65 or 66  67 and 68 and 69  limit 70 to (english language and yr="2013 -Current") |
| **Global Health** | Prevalen*.mp.  inciden*.mp.  morbidity.mp. or morbidity/  mortality.mp. or mortality/  burden of disease.mp.  disease burden.mp.  epidemiolog*.mp.  dementia/ or dementia.mp.  alzheimer.mp. or Alzheimer's disease/  latin america.mp. or Latin America/  caribbean.mp. or Caribbean/  Anguilla.mp. or Anguilla/  (Antigua and barbuda).mp. [mp=abstract, title, original title, broad terms, heading words, identifiers, cabicodes]  Aruba.mp. or Aruba/  bahamas.mp. or Bahamas/  Barbados/ or barbados.mp.  bonaire.mp. or Bonaire/  (Sint Eustatius and Saba).mp. [mp=abstract, title, original title, broad terms, heading words, identifiers, cabicodes]  British Virgin Islands.mp. or British Virgin Islands/  Cayman Islands.mp. or Cayman Islands/  Cuba.mp. or Cuba/  Curacao.mp. or Curacao/  Dominica/ or Dominica.mp.  Dominican Republic.mp. or Dominican Republic/  Grenada.mp. or Grenada/  Guadeloupe.mp. or Guadeloupe/  Haiti.mp. or Haiti/  Jamaica.mp. or Jamaica/  Martinique.mp. or Martinique/  Montserrat.mp. or Montserrat/  Puerto Rico.mp. or Puerto Rico/  Saint Barthelemy.mp. or Saint Barthelemy/  (Saint Kitts and Nevis).mp. [mp=abstract, title, original title, broad terms, heading words, identifiers, cabicodes]  Saint Lucia.mp. or Saint Lucia/  Saint Martin.mp. or Saint Martin/  (Saint Vincent and the Grenadines).mp. [mp=abstract, title, original title, broad terms, heading words, identifiers, cabicodes]  Sint Maarten.mp. or Sint Maarten/  (Trinidad and Tobago).mp. [mp=abstract, title, original title, broad terms, heading words, identifiers, cabicodes]  (Turks and Caicos).mp. [mp=abstract, title, original title, broad terms, heading words, identifiers, cabicodes]  United States virgin islands.mp. or United States Virgin Islands/  Belize.mp. or Belize/  Costa Rica.mp. or Costa Rica/  El Salvador.mp. or El Salvador/  Guatemala.mp. or Guatemala/  Honduras.mp. or Honduras/  Mexico/ or Mexico.mp.  Nicaragua.mp. or Nicaragua/  Panama.mp. or Panama/  Argentina.mp. or Argentina/  Bolivia.mp. or Bolivia/  Bouvet Island.mp.  Brazil/ or Brazil.mp.  Chile.mp. or Chile/  Colombia.mp. or Colombia/  Ecuador.mp. or Ecuador/  Falkland Islands.mp. or Falkland Islands/  French Guiana.mp. or French Guiana/  Guyana.mp. or Guyana/  Paraguay/ or Paraguay.mp.  Peru/ or Peru.mp.  (South Georgia and the South Sandwich Islands).mp. [mp=abstract, title, original title, broad terms, heading words, identifiers, cabicodes]  Suriname.mp. or Suriname/  Uruguay/ or Uruguay.mp.  Venezuela.mp. or Venezuela/  1 or 2 or 3 or 4 or 5 or 6 or 7  8 or 9  10 or 11 or 12 or 13 or 14 or 15 or 16 or 17 or 18 or 19 or 20 or 21 or 22 or 23 or 24 or 25 or 26 or 27 or 28 or 29 or 30 or 31 or 32 or 33 or 34 or 35 or 36 or 37 or 38 or 39 or 40 or 41 or 42 or 43 or 44 or 45 or 46 or 47 or 48 or 49 or 50 or 51 or 52 or 53 or 54 or 55 or 56 or 57 or 58 or 59 or 60 or 61 or 62 or 63 or 64  65 and 66 and 67  limit 68 to (english language and yr="2013 -Current") |
| **LILACS** | (prevalen* OR inciden* OR epidemiolog* OR morbidity OR mortality OR "burden of disease" OR "disease burden") AND (dementia OR alzheimer*) AND ("Latin America" OR "Caribbean" OR lac OR cuba* OR brazil* OR chile* OR mexic* OR colombia* OR argentin*) AND (instance:"regional") AND ( db:("LILACS" OR "BDENF" OR "BRISA" OR "colecionaSUS" OR "BBO") AND la:("en") AND year_cluster:("2015" OR "2014" OR "2013" OR "2016" OR "2017" OR "2018")) |

### Supplementary Materials 2: Modified Joanna Briggs Institute (JBI) Critical Appraisal Checklist for Studies Reporting Prevalence Data

Consistent with our previous study (Poon et al, 2020), a modified version of the JBI Checklist was used. Whereas the original JBI checklist contains 9 items with binary responses (yes/no), the modified version below uses a three-point rating scale (2 = Excellent, 1 = Good, and 0 = Not available/Unclear/Poor). An overall quality score is calculated as the sum of the score for each item (min = 0, max = 18).

1. Was the sample frame appropriate to address the target population?

2 Nationally/ regionally representative sample

1 Locally representative (limited generalisability)

0 Unrepresentative of any population

2. Were study participants recruited in an appropriate way?

2 Random sampling AND detailed recruitment process

1 Consecutive sampling OR (random sampling AND unclear recruitment process)

0 Unspecified

3. Was the sample size adequate?

2 >1500

1 500-1500

0 <500

4. Were the study subjects and setting described in detail?

2 Detailed description

1 Limited description/ Unclear description

0 No description

5. Was data analysis conducted with sufficient coverage of the identified sample?

2 Detailed description of refusal to participate, loss to follow up and exclusion criteria

1 Limited description of refusal to participate, loss to follow up and exclusion criteria

0 Unspecified/ Unclear description

6. Were valid methods used for the identification of the condition?

(The use of diagnostic manual was part of the inclusion criteria for this review)

2 Use of well recognised diagnostic manual AND exclusion of differential diagnoses (e.g. depression)

1 Use of well recognised diagnostic manual

0 No use of diagnostic manual

7. Was the condition measured in a standard, reliable way for all participants?

2 The condition was measured in a standard AND reliable way for all participants

1 The condition was measured in a reliable way for all participants

0 The condition was not measured properly

8. Was there appropriate statistical analysis?

2 Both crude and adjusted (or weighted) prevalence were calculated

1 Only crude prevalence was calculated

0 Statistical analysis was not conducted properly

9. Was the response rate adequate, and if not, was the low response rate managed appropriately?

2 ≥80%

1 60-79%

0 <60%

### Supplementary Material 3: Non-English studies

While we were unable to conduct a full systematic review of Spanish and Portuguese publications that contain dementia estimates, we have reviewed the non-English dementia data cited in a recent review by Nitrini and colleagues published in 2020 titled: “*Current trends and challenges on dementia management and research in Latin America*”. In this paper, Nitrini and colleagues cited two systematic review of published data in English, Portuguese or Spanish. The first of these was published in 2009 (21). Studies cited in this paper were excluded based on the data being outside the scope of the current review. The second study was a comprehensive systematic review of population-based dementia studies in LAC countries by Sanchez and colleagues published in 2019 (22). Sanchez et al reviewed 6 databases (PubMed, Ovid, LILACS, Cochrane, Scielo and Google scholar) and identified 25 studies published over the 26-year period between 1991 and 2016. Only five of the 25 studies are within the study period of our current review. Of these, two were already evaluated in our own systematic review. They are: (i) Correa P, Souza C, Alves R. Prevalence of dementia in elderly clients of private Healthcare plan: A study of the FIBRA-RJ, Brazil. *Dement Geriatric Cogn Disord*. 2013;35:77–86; and (ii) Dozzi S, Nitrini R. Cognitive impairment in individuals with low educational level and homogeneous sociocultural background. *Dement Neuropsychol*. 2014;8:345–50. The study by Dozzi et al was excluded from our study due to its small sample size. The remaining three studies identified by Sanchez et al were excluded because the studies did not use internationally recognized criteria for the diagnosis of dementia (e.g. DSM). Instead, they used a case definition of “test neurologico anormal” (“abnormal neurological test”) which does not differential cognitive symptoms from dementia from other psychiatric conditions, in particular depression and Parkinson’s disease.

The narrative review by Nitrini et al (2020) did not include studies published in Portuguese (the official language of LAC’s largest country, Brazil). Several studies from Brazil published in English were cited in Nitrini et al (2020), but only one falls within the time period of the current review. That study overlaps with results of our own systematic review and has already been incorporated into our model. Nitrini et al.’s earlier review from 2009 included a search of studies in Portuguese, but did not find any to include in their analysis. In fact, of the 8 studies identified by Nitrini et al (2009), only 3 were in Spanish. Of these, two would have been excluded from analysis on the basis that they were conference abstracts instead of published papers with clear methodologies that allow quality assessment for systematic reviews. The final paper would have been excluded for a lack of internationally recognized case definition.

All of the above give us confidence that any non-English studies that we might have missed are likely to be restricted to Spanish studies published in the 2-year period between 2017 and 2018. For a rough estimate of how many studies we might have missed, we reviewed the 25 studies identified by Sanchez et al (2019) to work out the total number of Spanish studies over this 26-year period that fits our inclusion criteria (ignoring the criterion on the study period). Only 12 of the 25 studies had used a gold standard definition of dementia (13 discarded). Of these only 3 studies were published in Spanish. This gives an average of 0.12 study per year, and 0.24 studies for the period we had missed.

### Supplementary Materials 4: Quality assessment using the Joanna Briggs Institute (JBI) Critical Appraisal checklist

| **Item of**  **JBI checklist** | | **1** | **2** | **3** | **4** | **5** | **6** | **7** | **8** | **9** |  |
| --- | --- | --- | --- | --- | --- | --- | --- | --- | --- | --- | --- |
| **Study** | **Authors (Year)** | **Sample frame** | **Sampling method** | **Sample size** | **Study subject and setting description** | **Sufficient coverage** | **Outcome ascertainment** | **Outcome measurement** | **Statistical analysis** | **Response rate** | **Sum**  **(out of 18)** |
| **1** | **Davis et al. (2018)** | **2** | **2** | **2** | **2** | **1** | **2** | **2** | **1** | **1** | **15** |
| **2** | **Eldemire-Shearer et al.(2018)** | **2** | **2** | **0** | **2** | **2** | **2** | **2** | **1** | **2** | **15** |
| **3** | **Pedraza et al. (2017)** | **2** | **1** | **1** | **2** | **1** | **2** | **2** | **1** | **2** | **14** |
| **4** | **César et al. (2016)** | **2** | **2** | **1** | **2** | **1** | **2** | **1** | **1** | **2** | **14** |
| **5** | **Bartoloni et al. (2014)** | **1** | **1** | **2** | **2** | **2** | **2** | **2** | **1** | **1** | **14** |
| **6** | **Velázquez-Brizuela et al. (2014)** | **2** | **2** | **1** | **2** | **2** | **2** | **1** | **1** | **0** | **13** |
| **7** | **Correa Ribeiro et al.(2013)** | **1** | **2** | **1** | **2** | **2** | **2** | **2** | **2** | **2** | **16** |
| **-Each item was assigned 2 marks: 2-Excellent; 1-Good; 0-Not available/ Unclear/ Poor**  **-The maximum quality score achievable was 18 (2 X 9 items)**  **-Refer to Appendix 8 for specific details** | | | | | | | | | | | |

### Supplementary Materials 5: Results of the sensitivity test

| Age group | Age -specific prevalence from JAGS analysis |
| --- | --- |
| 60-69 | 1.82% |
| 70-79 | 6.38% |
| >80 | 19.11% |
